# A Potential Synbiotic Strategy for the Prevention of Type 2 Diabetes: *Lactobacillus paracasei* JY062 and Exopolysaccharide Isolated from *Lactobacillus plantarum* JY039

**DOI:** 10.3390/nu14020377

**Published:** 2022-01-16

**Authors:** Jiayuan Zhao, Lihan Wang, Shasha Cheng, Yu Zhang, Mo Yang, Ruxue Fang, Hongxuan Li, Chaoxin Man, Yujun Jiang

**Affiliations:** Key Lab of Dairy Science, Ministry of Education, College of Food Science, Northeast Agricultural University, Harbin 150030, China; zjy1873679077@yeah.net (J.Z.); wang_lihan@163.com (L.W.); 15512473157@163.com (S.C.); jessedevil@163.com (Y.Z.); yang1994mo@163.com (M.Y.); fangruxue0926@163.com (R.F.); hongxuan_li@yeah.net (H.L.)

**Keywords:** type 2 diabetes, exopolysaccharide, synbiotic, intestinal microorganism, intestinal barrier function, inflammation

## Abstract

The disturbance of intestinal microorganisms and the exacerbation of type 2 diabetes (T2D) are mutually influenced. In this study, the effect of exopolysaccharides (EPS) from *Lactobacillus plantarum* JY039 on the adhesion of *Lactobacillus paracasei* JY062 was investigated, as well as their preventive efficacy against T2D. The results showed that the EPS isolated from *L. plantarum* JY039 effectively improved the adhesion rate of *L. paracasei* JY062 to Caco-2 cells (1.8 times) and promoted the proliferation of *L. paracasei* JY062. In the mice experiment, EPS, *L. paracasei* JY062 and their complex altered the structure of the intestinal microbiota, which elevated the proportion of *Bifidobacterium*, *Faecalibaculum*, while inversely decreasing the proportion of *Firmicutes*, *Muribaculaceae, Lachnospiraceae* and other bacteria involved in energy metabolism (*p* < 0.01; *p* < 0.05); enhanced the intestinal barrier function; promoted secretion of the gut hormone peptide YY (PYY) and glucagon-like peptide-1 (GLP-1); and reduced inflammation by balancing pro-inflammatory factors IL-6, TNF-α and anti-inflammatory factor IL-10 (*p* < 0.01; *p* < 0.05). These results illustrate that EPS and *L. paracasei* JY062 have the synbiotic potential to prevent and alleviate T2D.

## 1. Introduction

Diabetes mellitus is a chronic inflammatory disease that exists widely in the world and seriously endangers health [1]. According to the diagnostic criteria of the World Health Organization and the American Diabetes Association, the incidence rate of adult diabetes in mainland China is as high as 12.8%. Diabetes has become an urgent public health problem in China and most developing countries [2]. Type 2 diabetes (T2D) is the most common type of diabetes, especially in adults, which is characterized by insulin resistance and/or insufficient insulin secretion [3]. Additionally, an increasing number of clinical and animal studies have focused on the phenomenon of prevalent intestinal microorganism (IM) disturbance in patients with T2D. This is mainly evident from the differences in the composition and dominant flora of the IM and is related to the type of diabetes [4,5]. The IM of T2D patients is mainly characterized by the following: an altered abundance ratio of *Firmicutes* to *Bacteroidetes*, absence of short chain fatty acids (SCFAs) producing strains and enrichment of lipopolysaccharide (LPS) secreting Gram-negative bacterial strains [6]. In general, bacteria constrained by the intestinal barrier cannot enter other tissues and organs of the host from the intestine, but the disordered gut flora may produce excessive amounts of harmful metabolites to damage the intestinal barrier function and eventually trigger systemic diseases [7].

Based on the understanding of the interaction between IM and T2D, targeted regulation of intestinal microbiota is expected to be an effective adjuvant therapy for T2D [8]. As of now, many methods targeting the modulation of gut flora are developed and partially applied, such as fecal microbiota transplantation (FMT), probiotics, prebiotics, synbiotics, bacteriophages, antibiotics, etc. [9]. These methods have been proved to be effective, but some of them have been exposed as security risks. For example, some of the bacteria that are not insufficiently recognized may cause immune rejection and uncertain complications [10]. The virulence factor encoded by phage in fecal transplantation is still a safety issue [11]. In addition, the effect of antibiotics on intestinal flora has been proven to be long-term damage [12]. Probiotics and prebiotics are generally considered safe, they have been widely used in food, medical treatment, animal feed and other aspects. Meanwhile, they are widely sourced, easily accessible and less costly.

Benefiting from the unique advantages of probiotics, they have been extensively studied in a variety of disease models including T2D. Moreover, the mechanisms for alleviating T2D have also been partially revealed. The ways in which the gut microbiota influence T2D mainly include: altering the host gut microecological structure, such as reducing the Gram-negative bacterial strains (LPS producing strains) and increasing the content of SCFAs producing strains [13,14], mediating the farnesoid X receptor (FXR) signaling to regulate the bile acid metabolism [15], regulating the secretion of intestinal hormones, such as peptide YY (PYY) and glucagon-like peptide-1(GLP-1) [16] and reducing intestinal permeability by strengthening the intestinal barrier function [17,18]. 

Based on the broad efficacy exhibited by probiotics in various types of disease (or prevention) models and the disturbance of gut microbes prevalent in patients, supplementation with added probiotics in the diet is gradually becoming a consensus for most [19]. Probiotics administered orally endure one of two ways: some merely pass through by intestinal peristalsis, but others colonize the gut permanently. Probiotics that can stably engraft in the gut are believed to exert beneficial effects on the host in terms of increasing the efficiency of metabolic activity and enabling durable modulation of the indigenous microbiota [20]. Therefore, improvement of bacterial adhesion is crucial for effective colonization and maintenance of stable probiotics. However, how to effectively supplement probiotics to enhance the effective colonization of probiotics in the intestine is still a difficult point, especially for exogenous probiotics [20]. A few studies have confirmed that some organic compounds and functional natural ingredients can specifically improve the adhesion of bacterial strains or stimulate the expression of intestinal cell adhesion proteins. For example, Harimawan et al. found that the exopolysaccharides (EPS) of *P. aeruginosa* and *B. subtillis* could enhance self-adhesion and participate in biofilm formation to avoid clearance [21]; Wang et al. reported that liposomes coated with bacterial s-layer proteins (isolated from *Lactobacillus helveticus*) significantly enhanced the adhesion of liposomes to the intestinal tract [22]; Zhang et al. reported a Cationic Polythiophene Derivative poly (PMNT) with quaternary ammonium groups as side chains could efficiently promote the initial adhesion and biofilm formation of beneficial bacteria in gut microbiota [23]; and Mendoza et al. investigated the adhesion changes of lactobacillus cultured in milk supplemented with lactophospholipin, and the results showed that lactophospholipin could enhance the adhesion of lactobacillus to Caco-2 cells by promoting the expression of lactobacillus adhesion-related genes EF-TU and Cnb [24].

*Lactobacillus plantarum* is a lactic acid bacterium found in nutritive-rich environments of food, as well as in animal and human mucosae [25]. EPS are important biological products produced by some lactic acid bacteria. In addition to their health benefits, EPS are well known in the food and dairy industry for their shelf-life enhancement properties and ability to enhance technical functionality [26]. In addition, EPS promote the adhesion of lactic acid bacteria to eukaryotic cells, such as plants and the human gut, thus obtaining nutrients [27]. EPS are related to biofilm formation and adhesion to solid surfaces. In the biofilm, EPS also play a vital role in sequestering essential cations, cellular recognition and host–pathogen interactions [28], while there are few studies on the adhesion effect of lactic acid bacteria from different species. 

In our previous study, we confirmed the hypoglycemic effect of *Lactobacillus paracasei* JY062 in T2D mice [29]. Therefore, in this study, EPS extracted from *Lactobacillus plantarum* JY039 were used to improve the intestinal adhesion of *L. paracasei* JY062 and enhance its prebiotic function. The correlation between intestinal health and the development of diabetes was the focus of our study, and we further analyzed the structure of intestinal microflora, intestinal barrier function and other indicators related to intestinal health.

## 2. Materials and Methods

### 2.1. Microorganism and Culture Conditions

Both *L. pantarum* JY039 and *L. paracasei* JY062 were isolated from traditional fermented dairy products in Tibet, China. The MRS medium was used for the growth of the organism.

### 2.2. Isolation and Purification of the EPS

The JY039 strain was cultivated in the MRS medium at 37 °C for 20 h under a static condition. After incubation, the isolation and purification of EPS were performed by a modified method of Zhou et al. [30]. Briefly, the culture was centrifuged at 4 °C to eliminate the strain cells (10,000× *g*, 15 min). Then, the fermentation broth was heated at 100 °C for 10 min to inactivate enzymes capable of degrading the EPS. After cooling to room temperature, trichloroacetic acid (80%, *w/v*) was added to the EPS solution at a final concentration of 10% overnight at 4 °C. Centrifugation (12,000× *g*, 10 min, 4 °C) was carried out to remove the precipitated proteins. The supernatant was treated with three folds volumes of 95% (*v/v*) cold ethanol and overnight at 4 °C to precipitate the EPS which was gained through centrifugation (12,000× *g*, 10 min, 4 °C). The precipitated EPS were dissolved in deionized water and dialyzed (MWCO 8–14 KDa) with deionized water at 4 °C for 48 h and the deionized water was replaced every six hours. The crude EPS solution was purified by DEAE-Sepharose Fast Flow column (Φ26 mm × 300 mm, GE Healthcare, Fairfield, CT, USA), the eluted solution was deionized water with a flow rate of 0.5 mL/min and lyophilized for preparation. 

### 2.3. Monitoring the Growth Curve of L. paracasei JY062

The EPS utilization capacity of *L. paracasei* JY062 was determined by a modified method [31]. Briefly, *L. paracasei* JY062 was cultured in the MRS medium containing 2% glucose and 2% EPS in the same inoculation ratio, respectively. Next, an automatic microbial growth curve analyzer (Bioscreen C, OY Growth Curves Ab Ltd., Turku, Finland) and dilution coating method were used to monitor the growth curve and viable count of *L. paracasei* JY062 under two different culture conditions.

### 2.4. In Vitro Cell Adhesion Experiment

The in vitro cell adhesion experiment was accomplished using Caco-2 cells and the fluorescein labeling method with modification [32]. The EPS039 were dissolved in DMEM high glucose culture fluid (GIBCO, Invitrogen Corporation, Waltham, MA, USA) at 0, 10, 20, 30, 40 and 50 mg/mL, respectively. Freshly grown *L. paracasei* JY062 were labeled with 100 μM 5(6)-Carboxyfluorescein diacetate N-succinimidyl ester (cFDA-SE) (Sangon Biotech, Shanghai, China) in 1 × PBS (37 °C, 30 min), and washed twice with PBS. Then, the fluorescently labeled strains were suspended in different concentrations of EPS solutions, and the concentration of *L. paracasei* JY062 was adjusted to 10^9^ cfu/mL. The fluorescence spectrophotometer (Shimadzu, Kyoto, Japan) was selected to detect the initial fluorescence value (F_0_) of *L. paracasei* JY062, Ex/Em = 490/520 nm. Cells in DMEM supplemented with 10% heat-inactivated fetal bovine serum and 1% penicillin-streptomycin (GIBCO, Invitrogen Corporation, Waltham, MA, USA) were seeded in 6-well plates and cultivated until reaching a confluent state. Cells were washed three times with 1 × PBS, followed by incubation with 500 µL cFDA-SE labeled JY062 for 2 h at 37 °C. Next, the bacteria that did not adhere to the cells were sucked out and 300 µL trypsin was added to remove the cells and mixed, then the fluorescence value of cell/JY062 suspension (F_1_) was measured.

$$\mathrm{Adhesion}\;\mathrm{ratio}(\%)=\frac{F1}{F0}\times 100\%$$

### 2.5. Animals and Treatments

Fifty SPF C57BL-6J male mice (5 weeks, 20 ± 1 g) were purchased from Charles River Laboratories (Beijing, China). Animals were reared in a house at 23 ± 1 °C and 55 ± 5% humidity. The mice maintained in a 12-h light/dark cycle with ad libitum access to food and water. This study was approved by the Laboratory Animal Welfare and Ethics Committee of Northeast Agricultural University (Approval Number: NEAU-2020-09-0386-05). After one week of adaptive feeding, the mice were randomly divided into 5 groups (*n* = 10 per group): normal control (NC) group, diabetic (DM) group, *L. paracasei* JY062 protection (JY062) group, EPS + *L. paracasei* JY062 protection (EPP) group and EPS protection (EPS) group. Mice in the normal control group (NC) were treated with a normal chow diet; the DM, JY062, EPP and EPS groups were offered HFD consisting of 17% lard, 20% sucrose, 2.5% cholesterol, 0.5% sodium cholate and 60% normal diet. Meanwhile, the JY062, EPS and EPP group mice were fed extra complement with 0.2 mL of *L. paracasei* JY062 (1 × 10^9^ cfu/mL in skimmed milk), 0.2 mL of EPS solution (30 mg/mL in skimmed milk) and a mixed skim milk solution of EPS and *L. paracasei* JY062 (0.2 mL), respectively. The NC and DM group mice were administered 0.2 mL of skimmed milk. Body weight and blood glucose levels were recorded weekly. At the end of week 4, the DM, JY062, EPP and EPS group mice were intraperitoneally injected with streptozocin (STZ) (30 mg/kg body weight) (Sigma, St Louis, MO, USA), whereas the NC group rats received equal amounts of citric acid buffer alone. The fasting blood glucose (FBG) levels of all the mice were recorded at the end of week 5: the FBG of the DM group was higher than 11.1 mmol/L, indicating that the model was established successfully. At the end of week 12, fresh feces and intestinal contents were collected from the mice for the intestinal microbiota analyses. Meanwhile, blood serum, colons, the pancreas and liver were harvested and stored at −80 °C. Mice were anesthetized by an intraperitoneal injection of ketamine and diazepam before slaughter. We have listed the diet details in Table 1.

### 2.6. Biochemical Parameter Analysis

Blood was collected from mice via the tip of the tail from week 1 to week 12. Fasting blood glucose (FBG) was measured every week for 12 weeks and an oral glucose tolerance test (OGTT, 2 g/kg) was performed in the morning at the end of the 12th week. The area under the curve of glucose values (AUC-glucose) was calculated (0–120 min). The level of insulin, haemoglobin A1c (HbA1c), free fatty acid (FFA), lipopolysaccharide (LPS), IL-6, IL-10, TNF-α, peptide YY (PYY), glucagon-like peptide-1(GLP-1), triglyceride (TG), total cholesterol (TC), high-density lipoprotein cholesterol (HDL-C) and low-density lipoprotein cholesterol (LDL-C) were determined by enzyme-linked immunosorbent assay (ELISA) kits (Sigma, St Louis, MO, USA).

### 2.7. Intestinal Microbiota Analysis

The genomic DNA from the fecal samples was extracted by using the DNA extraction kit (NucleoSpin, MN, Wiesbaden, Germany). Next, the bacterial V3–V4 hypervariable regions of the 16S rRNA gene with the universal primers of 338F (5′-ACTCCTACGGGAGGCAGCA-3′) and 806R (5′-GGACTACHVGGGTWTCTAAT-3′) were magnified. DNA was purified by nucleic acid purification column and recovered from agarose gel. Then, the original data were spliced, and the spliced sequences were quality-filtered to remove chimeras and obtained high-quality Tags sequences. Finally, the diversity of the sequences was analyzed.

### 2.8. Hematoxylin and Eosin (H&E) Staining and Immunohistochemistry

The colon, liver and pancreas tissues were collected, and paraffin embedded. The tissues were sectioned to 4 μm thickness and then stained with hematoxylin and eosin (HE) for light microscopy examinations. Subsequently, an immunofluorescence analysis of insulin in the pancreas and an immunohistochemical analysis of claudin-1, Occludin-1, ZO-1 and Mucin-2 in the colon were performed.

### 2.9. Quantitative Real-Time PCR (RT-PCR)

RNA was isolated from the colon using Simply P Total RNA extraction kit (Bioer Technology Co., Hangzhou, China). The total RNA was used to synthesize cDNA with a reverse transcription kit (Takara Biomedical Technology Co., Beijing, China). The amplification and mRNA levels were determined by quantitative real-time PCR. The relative expression of mRNA was calculated using the 2^−ΔΔCt^ method. The primers used in this study are listed in Table 2.

### 2.10. Statistical Analyses

The data were presented as mean ± standard deviation (mean ± SD). The statistical analyses of groups were compared with a one-way analysis of variance (ANOVA). Significant differences and highly significant differences were indicated as *p* < 0.05 and *p* < 0.01. All statistical data analyses were performed with SPSS 25.0.

## 3. Results

### 3.1. Effect of EPS on the Growth of L. paracasei JY062

**Figure 1:**
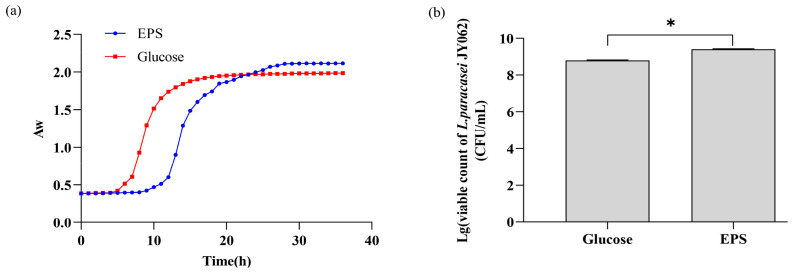
(**a**) Growth curves of *L. paracasei* JY062 in medium supplemented with glucose and EPS (2%), respectively; (**b**) viable count of *L. paracasei* JY062 in EPS medium and glucose medium. * *p* < 0.05 as compared to glucose.

Figure 1a shows the growth curves of *L. paracasei* JY062 in a medium supplemented with glucose and EPS, respectively. As can be seen from Figure 1a, *L. paracasei* JY062 had a longer lag phase in the medium supplemented with EPS (9 h) compared with the medium supplemented with glucose (5 h). Accordingly, the growth time of *L. paracasei* JY062 in the EPS medium to the end of exponential was also prolonged. We subsequently examined the viable count of *L. paracasei* JY062 grown to the end of exponential in different carbon sources (Figure 1b). It can be seen that the maximum viable count of *L. paracasei* JY062 in the EPS medium reached 2.55 × 10^9^ cfu/mL, while the maximum viable count was 6.30 × 10^8^ cfu/mL in the glucose medium. These results indicate that EPS could be utilized by *L. paracasei* JY062 and had the property that promoted the proliferation of *L. paracasei* JY062.

### 3.2. Effect of EPS on the Intestinal Adhesion Capacity of L. paracasei JY062

**Figure 2:**
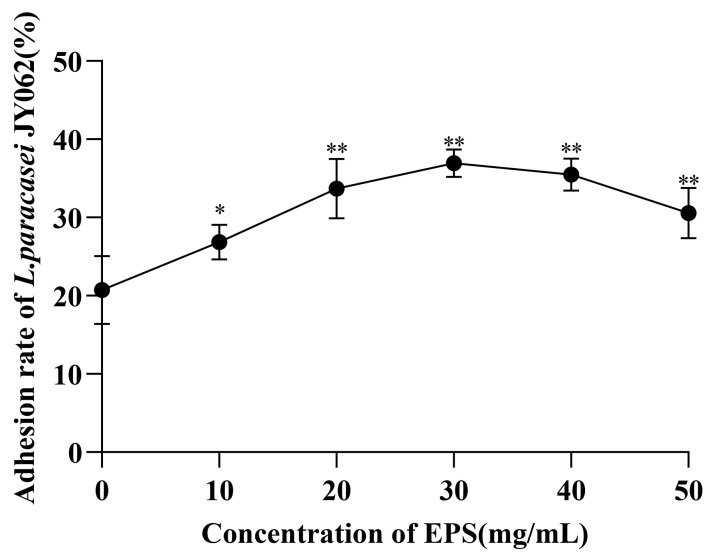
Effect of EPS concentration on the adhesion rate of *L. paracasei* JY062. (* *p* < *0.05,* ** *p* < 0.01).

The initial adhesion rate of *L. paracasei* JY062 to Caco-2 cells and the effect of different concentrations of EPS on the adhesion rate were measured through the fluorescent labeling method (Figure 2). With the increase of EPS concentration, the adhesion rate of *L. paracasei* JY062 to Caco-2 cells showed a trend of gradually increasing and then decreasing. It can be seen that the initial adhesion rate of *L. paracasei* JY062 was 20.72 ± 3.55% (means ± SD %), and when the concentration of EPS was added at 30 mg/mL, the adhesion rate increased to 36.94 ± 1.27% (means ± SD %). When the EPS concentration exceeded 30 mg/mL, the adhesion rate began to decrease. In conclusion, the addition of EPS significantly improved the adhesion rate of *L. paracasei* JY062 (*p* < 0.01; *p* < 0.05). This indicates that the adhesion rate of *L. paracasei* JY062 was enhanced by EPS and the adhesion rate was affected by the concentration of EPS.

### 3.3. Effects of Dietary Intervention on Blood Glucose and Lipid-Related Parameters

**Figure 3:**
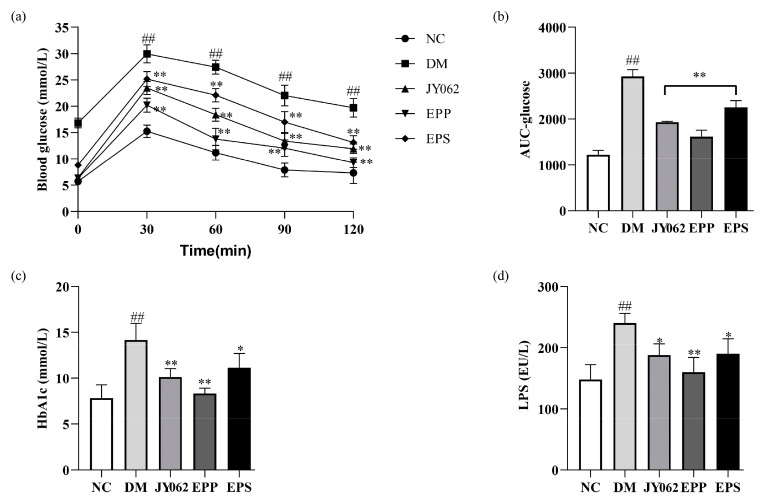
Effects of the dietary intervention on the biochemical parameters. (**a**) The glucose tolerance test (blood glucose values for 0–120 min); (**b**) area under the curve (AUC) values; (**c**,**d**) the levels of HbA1c and LPS were determined by ELISA kits. Data are presented as mean ± SD values. ## *p* < 0.01 vs. NC group; * *p* < 0.05, ** *p* < 0.01 vs. DM group.

Table 3 shows the fasting blood glucose (FBG) of the five groups from week 4 to week 12. It can be seen that after four weeks of different feeding, the FBG in JY062, EPP and EPS groups were significantly lower than that in the DM group (*p* < 0.01; *p* < 0.05). After the injection of STZ, the FBG in the DM, JY062, EPP and EPS groups all increased in week 5, but the DM group had a significantly higher FBG than the other four groups (*p* < 0.01; *p* < 0.05). From week 6 to week 12, the FBG was continuously increased in the DM group, while it was gradually decreased and significantly lower in the JY062, EPP and EPS groups than in the DM group (*p* < 0.01). Next, the changes in blood glucose levels among the groups from 0 min to 120 min are shown in Figure 3a. The blood glucose levels in the DM group were significantly higher than in the NC group from 0 to 120 min (*p* < 0.01). Furthermore, the blood glucose levels of the JY062, EPP and EPS groups were significantly lower than that in the DM group at different times (*p* < 0.01; *p* < 0.05). The AUC, HbA1c and LPS levels in the DM group were also significantly higher than those in the NC group (*p* < 0.01). The levels of AUC, HbA1c and LPS were significantly decreased in the JY062, EPP and EPS groups (*p* < 0.01; *p* < 0.05) (Figure 3b–d). In addition, high levels of TG, TC, FFA, LDL-C and insulin were detected in the DM group which were markedly reduced in the JY062, EPP and EPS groups (*p* < 0.05). However, the HDL-C content of the DM group was significantly lower than that in the NC group, and among the three dietary intervention groups, only the EPP group significantly increased the level of HDL-C (Table 4). These results show that either *L. paracasei* JY062 or EPS could improve the blood glucose and lipid metabolism in mice. Meanwhile, the combination of *L. paracasei* JY062 and EPS provided the best results.

### 3.4. The Impact of Dietary Intervention on Intestinal Microbiota Structure

**Figure 4:**
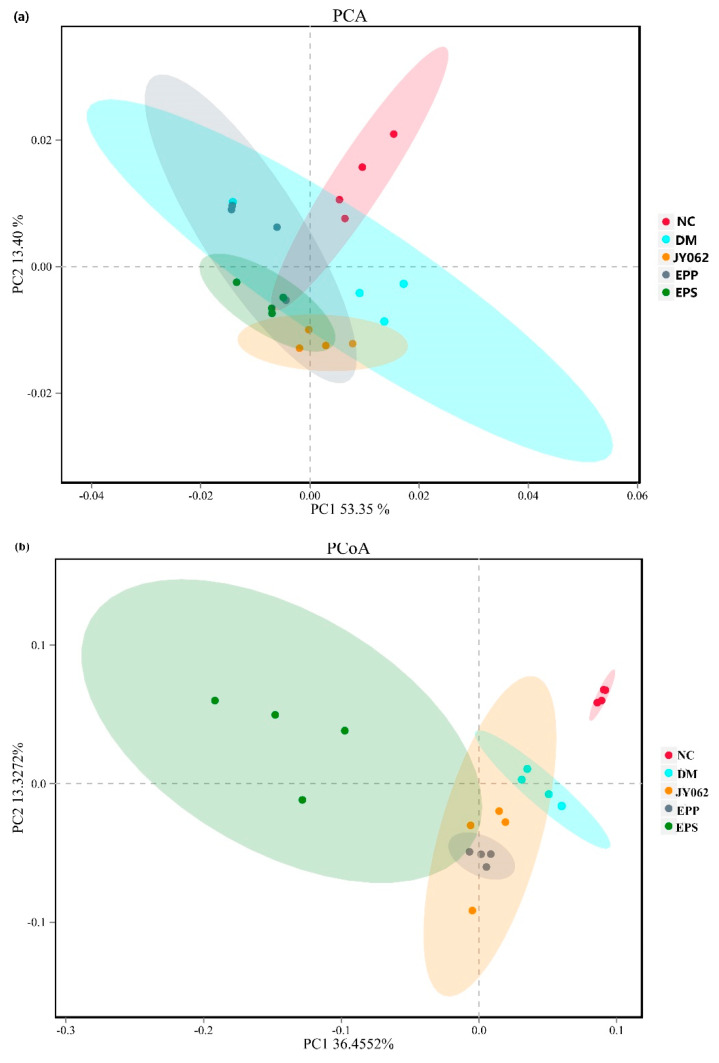

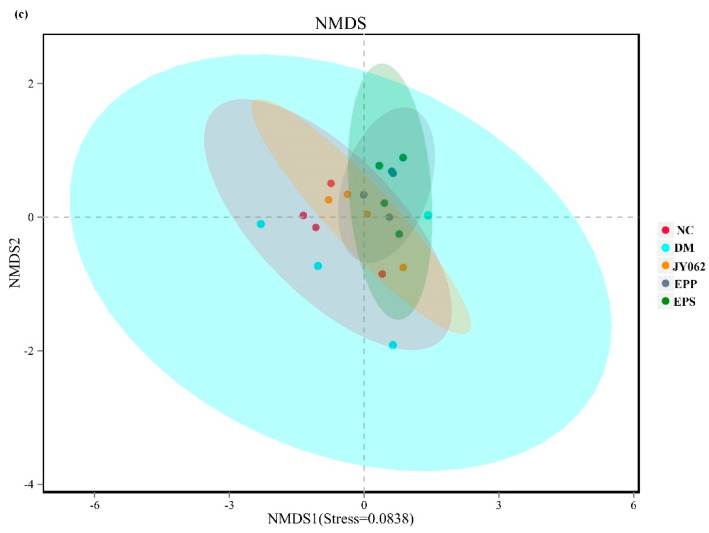
Differences in intestinal microbiome structure among the five groups (*n* = 4). Beta diversity analysis: (**a**) principal component analysis-PCA; (**b**) principal coordinates analysis-PCoA; (**c**) non-metric multidimensional scaling-NMDS.

We investigated the microbiota structure of intestinal contents in each group to explore the effects of different dietary interventions on intestinal microbiota. A beta diversity analysis was used to compare the similarity of the samples in species diversity, mainly including the principal component analysis (PCA), principal coordinates analysis (PCoA) and non-metric multidimensional scaling (NMDS) (Figure 4a–c). The results showed that the intestinal microbiota similarity of the DM group was significantly different from that of the NC group and the three dietary intervention groups. PCA, PCoA and NMDS depicted a visible separation between the NC, JY062, EPP, EPS and DM groups of mice.

### 3.5. The Impact of Dietary Intervention on Intestinal Microbiota Composition

**Figure 5:**
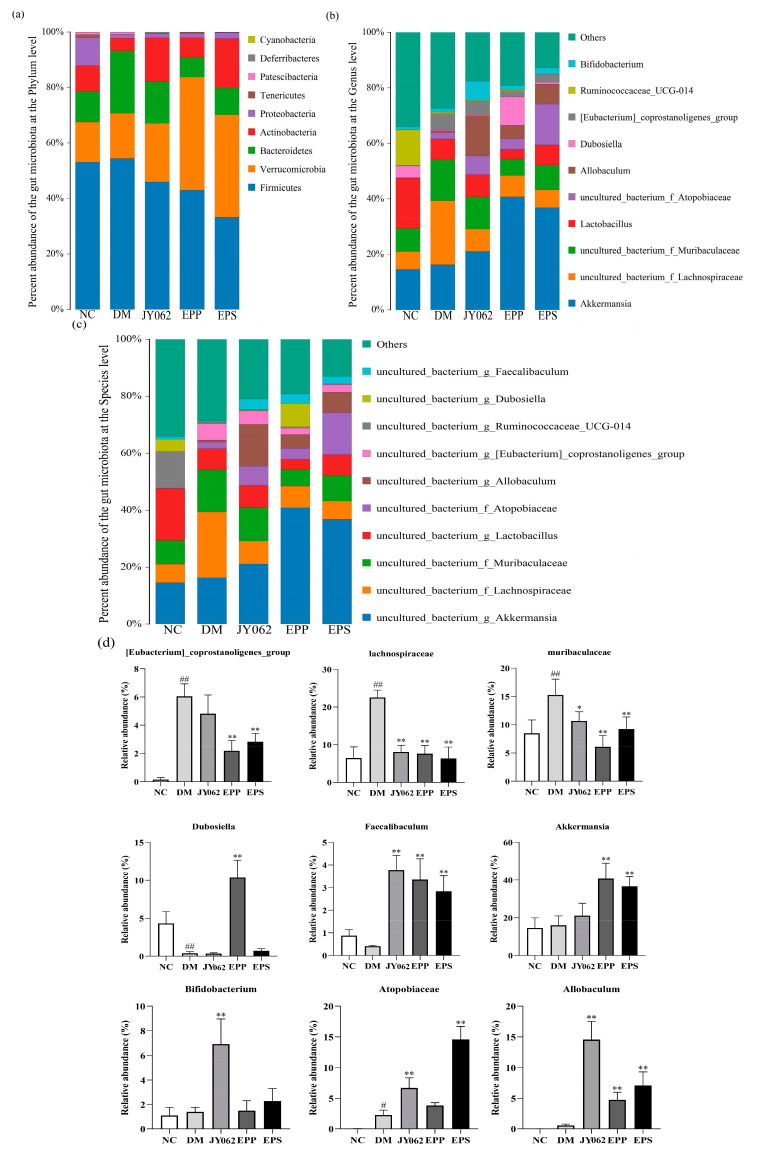

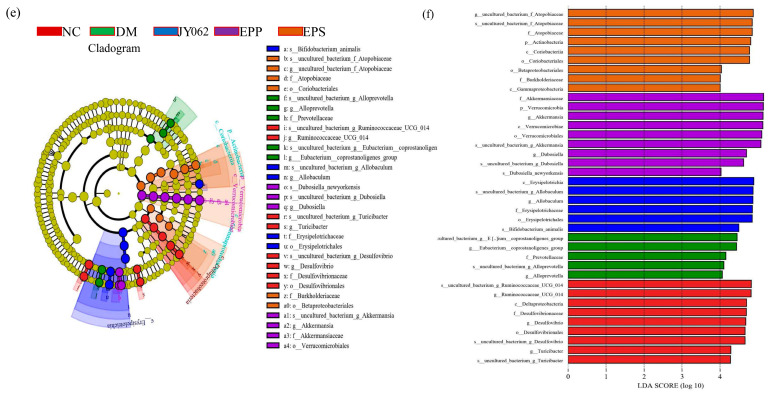
Differences in intestinal microbiota composition among the five groups (*n* = 4). (**a**) Differences in the abundance of intestinal microbiota at the phylum level; (**b**) differences in the abundance of intestinal microbiota at the genus level; (**c**) the relative abundance of intestinal microbiota at the species level is depicted; (**d**) the relative abundance of the intestinal microbiota with the greatest change in each group is depicted; (**e**) the LEfSe results of significant differential biomarkers among the 5 groups based on a cladogram; (**f**) the LEfSe results of significant differential biomarkers among the 5 groups based on LDA score (log10). Data are presented as mean ± SEM (*n* = 4), # *p* < 0.05, ## *p* < 0.01 vs. NC group; * *p* < 0.05, ** *p* < 0.01 vs. DM group.

Figure 5a shows the composition of intestinal flora at the phylum level: more than 97% of the gut bacteria in mice was dominated by *Firmicutes*, *Bacteroidetes*, *Verrucomicrobia*, *Actinobacteria* and *Proteobacteria* in the five groups. *Firmicutes* accounted for the largest proportion in the DM group (54.5%), slightly higher than the NC group (53.0%) and significantly higher than the three dietary intervention groups (*p* < 0.05). The proportion of *Bacteroidetes* in the DM group was also significantly higher than that in the NC group and three dietary intervention groups (*p* < 0.05). On the contrary, the proportion of *Actinobacteria* and *Proteobacteria* in the DM group was significantly lower than that in the NC group (*p* < 0.05, *p* < 0.01). We also noted that these changes were reversed to varying degrees in the EPS, JY062 and EPP groups. In addition, it was noteworthy that the proportion of *Verrucomicrobia* in the three dietary intervention groups increased significantly compared to the NC group and the DM group. At the genus level and species level (Figure 5b,c), compared with the NC group, the abundance of *[Eubacterium]_coprostanoligenes_group*, *Muribaculaceae* and *Lachnospiraceae* in the DM group increased significantly, but decreased significantly in the three dietary intervention groups (*p* < 0.05, *p* < 0.01). In addition, *Bifidobacterium* (JY062), *Dubosiella* (EPP), *Allobaculum*, *Atopobiaceae* and *Akkermansia* were significantly enriched in the EPS, JY062 and EPP groups compared with the DM group and NC group (*p* < 0.01). Furthermore, *Faecalibaculum* was significantly enriched in the EPS, JY062 and EPP groups compared with the NC group and DM group (Figure 5d, *p* < 0.01).

An LEfSe (line discriminant analysis (LDA) effect size) analysis was then used to find biomarkers with statistical differences. As depicted in Figure 5e, different specific bacterial taxa were enriched in the five groups. Figure 5f shows the species whose LDA score was greater than the set value (4.0). The length of the bar chart represents the impact size of different species. It can be seen that the species with significant differences among the groups were obviously different. Compared with other groups, there were fewer species with significant differences in the DM group and the impact was lower.

### 3.6. The Impact of Dietary Intervention on Intestinal Barrier Function

**Figure 6:**
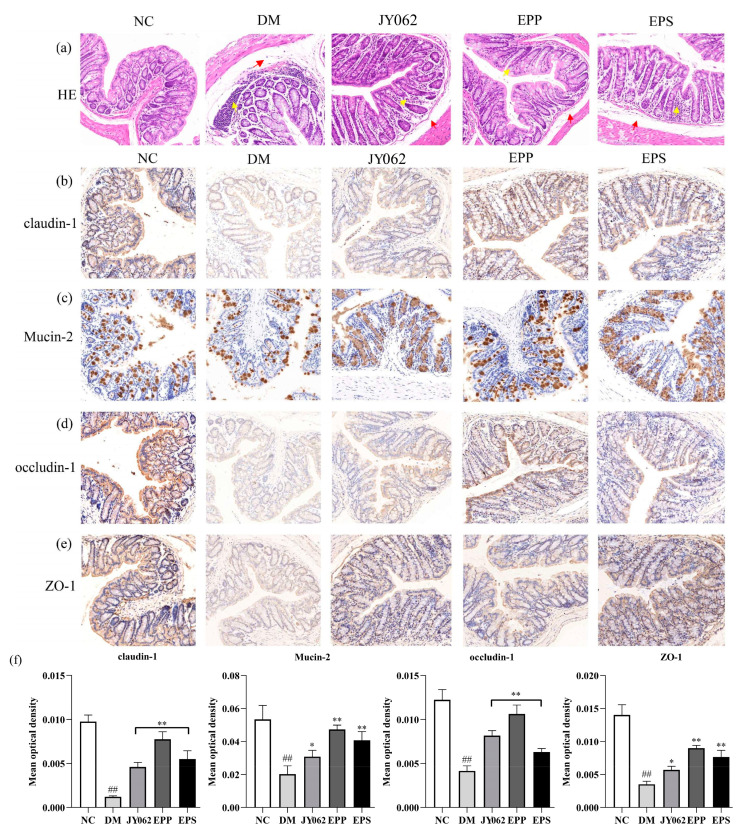
Effects of dietary intervention on intestinal barrier function. (**a**) The colonic microsections stained with hematoxylin-eosin (HE) (200× magnification); the protein expression of claudin-1 (**b**), Mucin-2 (**c**), occludin-1 (**d**) and ZO-1 (**e**) determined by immunohistochemistry (original magnification 200×); (**f**) the mean optical density of claudin-1, Mucin-2, occludin-1 and ZO-1. Data are presented as mean ± SD values. ## *p* < 0.01 vs. NC group; * *p* < 0.05, ** *p* < 0.01 vs. DM group.

The intestinal barrier is an important protection for a healthy metabolic environment, and its function was also one of the focuses of our research. Figure 6a shows HE histological sections of the colon tissue. In the DM group, the epithelial and mucosa structures were visibly damaged, with fewer goblet cells in the mucous layer, severe mucosal edema (as indicated by the red arrows) and massive inflammatory cells infiltration (as indicated by the yellow arrows). However, the EPS, JY062 and EPP groups reduced these symptoms to varying degrees. A further immunohistochemical analysis revealed that the levels of claudin-1, Mucin-2, occludin-1 and ZO-1 were lower in the DM group than in the NC group (Figure 6b–e). Figure 6f shows the mean optical density values of these four tight junction proteins. Mice treated with EPS, *L. paracasei* JY062 and EPP showed elevated protein expression compared with the DM group (*p* < 0.05, *p* < 0.01). 

### 3.7. The Impact of Dietary Intervention on Liver and Pancreas Function 

**Figure 7:**
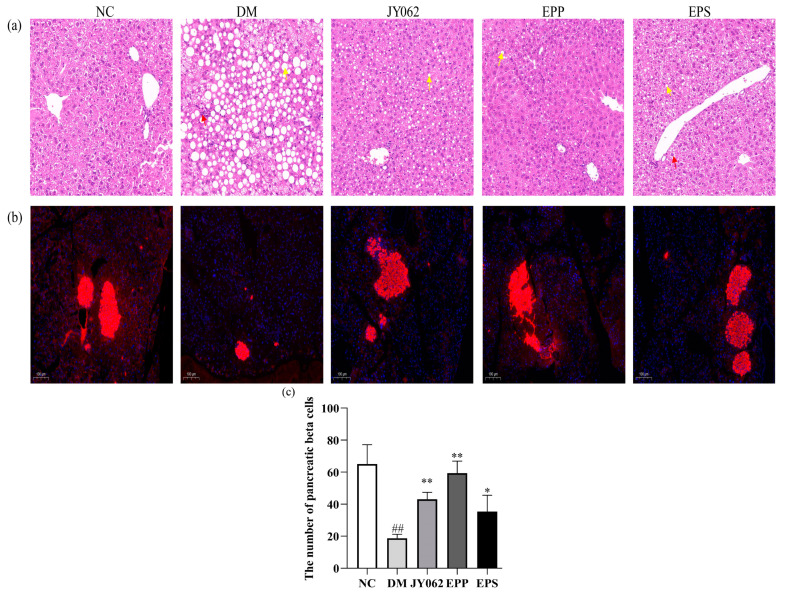
Effects of dietary intervention on liver and pancreas function. (**a**) The liver microsections were stained with hematoxylin-eosin (HE) (100× magnification); (**b**) immunofluorescence of insulin in pancreatic tissue; (**c**) the number of pancreatic beta cells. Data are presented as mean ± SD values. ## *p* < 0.01 vs. NC group; * *p* < 0.05, ** *p* < 0.01 vs. DM group.

The main role of the pancreas is to secrete insulin and glucagon, while the liver is one of the most important insulin receptor organs. Therefore, the HE pathological analysis was performed on the liver (Figure 7a). In the DM group, the liver tissue structure was severely abnormal, with a large area of hepatic cell steatosis, a large number of fat droplets of different sizes (as indicated by the yellow arrows) and inflammatory cell infiltration in the liver parenchyma (as indicated by the red arrows). However, in the EPS, JY062 and EPP groups, the degree of hepatic steatosis was significantly lower than that in the DM group, and no significant inflammatory infiltration was observed. Figure 7b shows an immunofluorescence analysis of insulin (the red part of the picture) in the pancreas. Figure 7c shows the difference in the number of pancreatic beta cells, and the number of pancreatic beta cells in the DM group was significantly lower than that in the other four groups (*p* < 0.05, *p* < 0.01). 

### 3.8. The Impact of Dietary Intervention on Gastrointestinal Hormones and Inflammatory Factors

**Figure 8:**
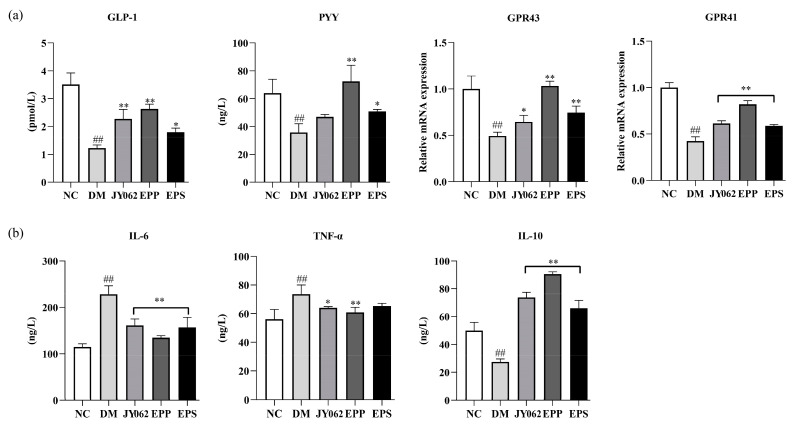
Effects of dietary intervention on gastrointestinal hormones and inflammatory factors. (**a**) The contents of GLP-1 and PYY in serum (determined by Elisa kits) and the mRNA expression of GPR41/43 in colon tissue (determined by RT-PCR); (**b**) the contents of TNF-α, IL-6 and IL-10 in serum (determined by ELISA kits). Data are presented as mean ± SD values. ## *p* < 0.01 vs. NC group; * *p* < 0.05, ** *p* < 0.01 vs. DM group.

Intestinal hormones are a kind of hormones secreted by intestinal cells, which can regulate intestinal metabolism and play an important role in regulating appetite, blood sugar and intestinal function. The serum levels of PYY and GLP-1 in each group were detected, and the mRNA expression of GPR41/43 on which the secretion of these two hormones depends (Figure 8a). The levels of PYY, GLP-1 and the mRNA levels of GPR43, GPR41 in the DM group were significantly lower compared to the NC group (*p* < 0.01). However, compared with the DM group, the content and expression of PYY, GLP-1, GPR41 and GPR43 in the other three treatment groups were increased to varying degrees. We further explored the effects of dietary intervention on the expression of inflammatory factors. The levels of TNF-α, IL-6 and IL-10 in blood serum were detected (Figure 8b). It can be seen that compared with the NC group, the levels of pro-inflammatory factors TNF-α and IL-6 in the DM group were significantly increased, and the level of anti-inflammatory factor IL-10 was significantly decreased. However, mice treated with EPS, *L. paracasei* JY062 and EPP reversed the trend of the DM group (*p* < 0.05, *p* < 0.01). 

## 4. Discussion

T2D is a chronic, progressive disease that develops as a result of an unhealthy diet [33]. Therefore, it is necessary and valuable to take intervention measures in daily diets to prevent or alleviate T2D. In this study, we examined a dietary intervention that approximates symbiosis. As a carbon source, the EPS isolated from *L. plantarum* JY039 could be degraded by *L. paracasei* JY062 with hypoglycemic function, and the viable number of *L. paracasei* JY062 was significantly increased. It suggests that the EPS have potential prebiotic properties for increasing *L. paracasei* JY062. The reason for the prolonged lag-phase within the EPS medium was assumed to be that the bacteria has to secret more degrading enzymes to hydrolyze the large molecular EPS into smaller oligosaccharides for further digestion in vivo, but it could uptake glucose directly [31].

Enhancing the adhesion of probiotics is one of the effective ways to achieve effective colonization. In this study, EPS isolated from *L. plantarum* JY039 effectively enhanced the adhesion rate of *L. paracasei* JY062 to Caco-2 cells. Prior to this, only a few prebiotics have been reported to act as a connector between probiotics and host intestinal cells, and most prebiotics have no effect on probiotics adhesion, or even competitively inhibit probiotics adhesion [34,35,36]. To our knowledge, this is the first time that EPS have been shown to enhance the adhesion of lactic acid bacteria of different species’ origin. Moreover, the adhesion rate was affected by the concentration of EPS. This may be caused by the fact that the EPS from lactic acid bacteria have a certain inhibitory effect on cancer cells [37]. Furthermore, we observed cell detachment at concentrations exceeding 30 mg/mL, whereas, the reason why EPS enhance the adhesion of *L. paracasei* JY062 in this study remains unclear.

The detection results of metabolites in serum showed that EPS, *L. paracasei* JY062 and EPP could reduce the factors inducing T2D to varying degrees, such as LPS, FFA, TG, TC, LDL-C and other indicators. The LPS is mainly secreted by Gram-negative bacteria and is observed to be significantly increased in many studies of T2D and inflammatory diseases [38,39]. LPS can permeate intestinal epithelial cells and bind to chylomicrons, which are subsequently transported to insulin sensitive organs, eventually causing inflammation and insulin resistance [40]. Low-grade chronic inflammation is one of the main features of T2D, and patients often accumulate excessive inflammatory markers [41]. In this study, the results showed that the levels of pro-inflammatory factors such as IL-6 and TNF-α were decreased in the EPS, EPP and JY062 groups, while the levels of anti-inflammatory factors IL-10 were increased. Wang et al. reported that such changes in inflammatory factors may be through modifying the M1/M2 phenotype macrophage [16]. The levels of these pro-inflammatory factors were positively correlated with LPS and FFA. Previous studies have shown that LPS-induced inflammation is one of the causes of pancreatic beta cell dysfunction [42], which also explains why the DM mice had the most severe inflammation and structural abnormalities of tissues. Liver steatosis in the DM group can be explained by the excess accumulation of these lipid metabolites [43]. 

The difference of intestinal microflora structure in each group has important reference significance to explain the different experimental results. At the phylum level, the *Firmicutes* and *Bacteroidetes* had the highest proportion in the DM group (nearly 80%), which was higher than that in the NC group and the EPS, EPP and JY062 groups. Previous studies on *Firmicutes* and *Bacteroidetes* pointed out that many members of the *Firmicutes* and *Bacteroidetes* can encode and secrete carbohydrate active enzymes that facilitate more efficient hydrolysis and utilization of carbohydrates [44]. Meanwhile, *Firmicutes* produce more absorbable energy from hydrolyzed carbohydrates than *Bacteroidetes* [45]. The results of this study showed that dietary intervention reduced the enrichment of *Firmicutes* and *Bacteroidetes* induced by a high-fat diet compared with the DM group, suggesting that dietary intervention may reduce the host’s absorption of energy by changing the composition of intestinal flora. A clinical study found that *Bacteroides* was significantly increased in patients with T2D compared with normal subjects [46]. This is consistent with our findings. It is worth noting that the proportion of *Verrucomicrobia* in the DM group did not change significantly compared with the NC group, but significantly increased in the EPS, EPP and JY062 groups. Zhang et al. reported that *Verrucomicrobia* may be a characteristic bacterium of T2D, as *Verrucomicrobia* was significantly reduced in the early and advanced stages of T2D patients [47]. Barcena et al. found that transplanting *Verrucomicrobia* bacteria to progeria mice delayed aging and increased lifespans [48]. We also observed large differences between groups in the proportion of *Actinobacteria*. The proportion of *Actinobacteria* was significantly decreased in the DM group compared with the NC group, whereas the other three dietary interventions increased the proportion of *Actinobacteria.* In some previous studies, the therapeutic effects of certain bacteria belonging to the *Actinobacteria* on gastrointestinal and systemic diseases were revealed, and although *Actinobacteria* do not account for a high proportion of several common dominant phyla, they are critical for maintaining intestinal homeostasis [49]. This result suggests that dietary interventions can maintain intestinal homeostasis by regulating the abundance of *Actinomycetes*. At the genus level, compared with the NC group and three dietary intervention groups, the proportion of *Muribaculaceae* and *Lachnospiraceae* in the DM group was significantly increased. In a previous study of lipid metabolism disorders in rats induced by a high-fat diet, a large increase in *Muribaculaceae* was also observed in the model group [50]. Zeng et al. reported that *Lachnospiraceae* significantly increased in mice with colitis induced by a long-term high-fat diet [51]. This suggests that these two genera of gut bacteria are closely related to host lipid metabolism disorders. In addition, *Bifidobacterium, Dubosiella, Allobaculum, Atopobiaceae, Akkermansia* and *Faecalibaculum* were enriched to varying degrees in the three dietary intervention groups. According to previous studies, they also have an explicit or potential association with gut microecological health [52,53,54,55]. This mainly includes the function of resisting LPS, enhancing intestinal mucosal function and producing SCFAs. In addition, the effect of EPS on the host gut flora is of concern. As can be seen from the results of the gut flora in the EPS, JY062 and EPP groups, compared with the JY062 group, the abundances of *[Eubacterium]_coprostanoligenes_group, Lachnospiraceae* and *Muribaculaceae* in the EPS and EPP groups were significantly reduced, while the abundances of *Dubosiella* and *Akkermansia* were significantly increased in the EPS and EPP groups. This suggests that EPS have unique properties in altering the gut flora. According to previous studies, the EPS of lactic acid bacteria have extensive effects on intestinal flora [56]. The EPS’ role as carbon reserve may have contributed to the proliferation of some gut bacteria as a nutrient substrate [57].

Intestinal microbiome disruption disrupts the physical and microbial barriers of the intestine, which in turn increases intestinal permeability and ultimately leads to inflammation and systemic disease [9]. The functions of tight junction proteins such as claudin-1, ZO-1 and mucin-2 are primarily to regulate intestinal barrier function and prevent luminal macromolecules (bacteria, toxins) from entering the bloodstream [58]. The immunohistochemical results of these proteins were significantly lower in the DM group than in the NC group and the EPS, EPP and JY062 groups, and the results of the HE pathological analysis also showed a consistent trend. These results reflect that dietary intervention alleviated T2D by maintaining the intestinal barrier function. It also explained that the increased number of harmful serum metabolites, abnormal organs and tissue structures in the DM group mice were caused by the impaired intestinal barrier function. GPR41/43 are two receptors for SCFAs (acetic acid and propionic acid) that are mainly responsible for the regulation of gut hormone secretion [9]. When intestinal microbiota is disrupted, SCFAs are reduced and the stimulation to GPR41 is also reduced, which leads to decreased secretion of insulin and PYY from pancreatic β cells and intestinal L-epithelial cells regulated by GPR41 [59,60]. Similarly, GPR43 is mainly involved in energy metabolism to maintain the energy balance through regulating the secretion of GLP-1 by intestinal L-epithelial cells [61]. Studies have shown that PYY can inhibit pathological overeating effectively by acting on the neuro-mediated hypothalamus. Moreover, it can improve the survival and function of islet cells in vivo, and has a certainly therapeutic effect on diabetes [62]. GLP-1 can promote insulin secretion by pancreatic β cells, inhibit glucagon secretion by pancreatic α cells and protect pancreatic β cells from glycotoxicity and other inflammatory damage [63]. Corresponding to this, in the DM group, the mRNA expression levels of GPR41 and GPR43 were significantly decreased, and the serum levels of PYY and GLP-1 were also decreased correspondingly. This suggests that it may be related to the activation of GPR41 / 43, but the secretion of intestinal hormones is a complex process. More experiments are needed to prove the correlation between GPR41 / 43 and the secretion of intestinal hormones. In addition, the content of insulin in the pancreatic tissue of the DM group also decreased. However, the EPS, EPP and JY062 groups showed an opposite trend to the DM group. 

## 5. Conclusions

Taken together, our study confirmed that the EPS extracted from *L. plantarum* JY039 have prebiotic potential to promote the proliferation of *L. paracasei* JY062, and also verified that the EPS can enhance the adhesion of *L. paracasei* JY062 to Caco-2 cells in vitro. Next, the preventive effects of *L. paracasei* JY062, EPS and EPS + *L. paracasei* JY062 on T2D were explored in mice. The results of this study showed that dietary interventions could improve intestinal microbiota by promoting SCFAs producing bacterial growth and inhibiting the LPS producing bacteria. Next, dietary interventions improved glucose and lipid metabolism and pancreas function by increasing the secretion of PYY and GLP-1. Moreover, dietary interventions reduced inflammation by modulating the balance of pro-inflammatory and anti-inflammatory factors. In addition, the dietary interventions enhanced the intestinal barrier function by up-regulating the levels of tight junction proteins. This study provides a novel approach for elevating the adhesion of a probiotic and a synbiotic strategy for improving T2D through dietary intervention. However, the intestinal environment of animals is extremely complex, and the colonization of the intestine by probiotics has great individual variability. Studying probiotic colonization in the gut requires more rigorous experimental designs, as well as large animal models.

## Figures and Tables

**Table 1:** Details of dietary treatment of mice.

Group	Treatment (Week 1–12)
NC	A normal chow diet
DM	High-fat diet (HFD)
JY062	HFD and 0.2 mL of *L. paracasei* JY062, daily
EPP(EPS + JY062)	HFD and 0.2 mL of the mixture (EPS and *L. paracasei* JY062), daily
EPS	HFD and 0.2 mL of EPS solution, daily

**Table 2:** Primer sequences used for RT-PCR.

Genes	Forward (5′–3′)	Reverse (5′–3′)
β-actin	CTACCTCATGAAGATCCTGACC	CACAGCTTCTCTTTGATGTCAC
GPR41	CCACATGCTCATCTTCTTCGTCTG	ACGGACTCTCAGGCTGACATAG
GPR43	CTGTATGGATGATCGCTGCTCTG	CTGCTCTTGGGTGAAGTTCTCGTAG

**Table 3:** Fasting blood glucose (FBG) of mice in the five groups from week 4 to week 12.

Time (week)	NC (mmol/L)	DM (mmol/L)	JY062 (mmol/L)	EPP (mmol/L)	EPS (mmol/L)
Week4	4.72 ± 0.25 ^a^	5.80 ± 0.43 ^b^	4.13 ± 0.40 ^ac^	3.53 ± 0.19 ^c^	4.73 ± 0.58 ^a^
Week5	4.70 ± 0.59 ^a^	13.27 ± 0.49 ^b^	9.83 ± 0.26 ^c^	7.83 ± 0.62 ^d^	12.10 ± 0.37 ^e^
Week6	4.53 ± 0.59 ^a^	14.50 ± 0.88 ^b^	8.73 ± 0.62 ^c^	7.20 ± 0.29 ^d^	11.30 ± 0.37 ^e^
Week7	4.97 ± 0.45 ^a^	15.13 ± 0.79 ^b^	8.20 ± 0.78 ^c^	6.97 ± 0.54 ^c^	10.10 ± 0.62 ^d^
Week8	5.53 ± 0.31 ^a^	15.80 ± 0.43 ^b^	7.93 ± 0.48 ^c^	6.23 ± 0.45 ^a^	10.80 ± 0.37 ^d^
Week9	4.77 ± 0.39 ^a^	16.83 ± 0.29 ^b^	7.63 ± 0.40 ^c^	6.57 ± 0.53 ^d^	9.67 ± 0.65 ^e^
Week10	4.67 ± 0.33 ^a^	16.33 ± 0.42 ^b^	7.93 ± 0.53 ^c^	6.70 ± 0.42 ^d^	9.70 ± 0.49 ^e^
Week11	4.87 ± 0.31 ^a^	16.67 ± 0.31 ^b^	7.80 ± 0.22 ^c^	6.93 ± 0.56 ^c^	9.60 ± 0.51 ^d^
Week12	5.70 ± 0.41 ^a^	16.80 ± 0.75 ^b^	7.87 ± 0.12 ^c^	6.30 ± 0.45 ^a^	9.13 ± 0.59 ^d^

Data are shown as mean ± SD (*n* = 10). Different superscripted letters in the same line are significantly different (*p* < 0.05).

**Table 4:** Effects of dietary intervention on serum FFA, TC, TG, HDL-C, LDL-C and insulin in mice.

Group	FFA (mmol/L)	TC (mmol/L)	TG (mmol/L)	HDL-C (mmol/L)	LDL-C (mmol/L)	Fasting Insulin (mIU/L)
NC	0.19 ± 0.03 ^a^	3.94 ± 0.25 ^a^	1.58 ± 0.07 ^a^	6.44 ± 0.76 ^a^	0.26 ± 0.14 ^a^	8.3 ± 1.1 ^a^
DM	0.52 ± 0.02 ^b^	6.13 ± 0.31 ^b^	2.89 ± 0.17 ^b^	3.55 ± 0.76 ^b^	1.18 ± 0.12 ^b^	17.0 ± 1.5 ^b^
JY062	0.30 ± 0.03 ^c^	4.66 ± 0.40 ^ac^	0.97 ± 0.20 ^c^	4.00 ± 0.33 ^b^	0.64 ± 0.10 ^d^	10.5 ± 0.9 ^ac^
EPP	0.21 ± 0.02 ^a^	4.42 ± 0.32 ^ac^	0.69 ± 0.10 ^c^	6.11 ± 0.40 ^a^	0.39 ± 0.04 ^ac^	9.1 ± 1.3 ^a^
EPS	0.35 ± 0.02 ^d^	4.80 ± 0.44 ^c^	1.07 ± 0.30 ^c^	4.79 ± 0.17 ^b^	0.59 ± 0.05 ^cd^	12.2 ± 1.1 ^c^

Data are shown as mean ± SD (*n* = 10). Different superscripted letters in the same column are significantly different (*p* < 0.05).

## Data Availability

Data in the project is still being collected, but all data used in the study is available by contacting the authors.
